# A Model of Malaria Epidemiology Involving Weather, Exposure and Transmission Applied to North East India

**DOI:** 10.1371/journal.pone.0049713

**Published:** 2012-11-27

**Authors:** Prashant Goswami, Upadhayula Suryanarayana Murty, Srinivasa Rao Mutheneni, Avinash Kukkuthady, Swathi Trithala Krishnan

**Affiliations:** 1 CSIR Centre for Mathematical Modelling and Computer Simulation [C-MMACS], Bengaluru, India; 2 Biology Division, CSIR-Indian Institute of Chemical Technology, Hyderabad, India; Kenya Medical Research Institute (KEMRI), Kenya

## Abstract

**Background:**

Quantitative relations between weather variables and malaria vector can enable pro-active control through meteorological monitoring. Such relations are also critical for reliable projections in a changing climate, especially since the vector abundance depends on a combination of weather variables, each in a given range. Further, such models need to be region-specific as vector population and exposure depend on regional characteristics.

**Methods:**

We consider days of genesis based on daily temperature, rainfall and humidity in given ranges. We define a single model parameter based on estimates of exposure and transmission to calibrate the model; the model is applied to 12 districts of Arunachal Pradesh, a region endemic to malaria. The epidemiological data is taken as blood samples that test positive. The meteorological data is adopted from NCEP daily Reanalysis on a global grid; population data is used to estimate exposure and transmission coefficients.

**Results:**

The observed annual cycles (2006–2010) and the interannual variability (2002–2010) of epidemiology are well simulated for each of the 12 districts by the model. While no single weather variable like temperature can reproduce the observed epidemiology, a combination of temperature, rainfall and humidity provides an accurate description of the annual cycle as well as the inter annual variability over all the 12 districts.

**Conclusion:**

Inclusion of the three meteorological variables, along with the expressions for exposure and transmission, can quite accurately represent observed epidemiology over multiple locations and different years. The model is potentially useful for outbreak forecasts at short time scales through high resolution weather monitoring; however, validation with longer and independent epidemiological data is required for more robust estimation of realizable skill. While the model has been examined over a specific region, the basic algorithm is easily applicable to other regions; the model can account for shifting vulnerability due to regional climate change.

## Background

Malaria continues to be a killer disease for many regions across the world; people living in remote areas away from adequate medical facilities are especially vulnerable. Outbreaks of malaria depend on the abundance of mosquito density, exposure of the human host to bites and the rate of transmission. However, for a given geographical location and population (and socio-economic practices) with certain immunological history, the abundance of the vectors can be said to determine the dynamics of the epidemic in terms of infection. It is worth emphasizing, however, that the toll of the epidemic, in terms of deaths, will also depend critically on several other factors like access to health care, especially in remote areas; statistical analysis of community-based epidemiological studies in remote and forested terrains in the north-east of India shows accessibility to the nearest health center as the primary risk factor [1]–[3]. However, pro-active vector sanitation can significantly reduce the risk of infection. In particular, identification of the peaks in vector population which would precede the disease outbreak by a typical incubation time of the parasite in the human host can enable pro-active control of malaria [4]. Vector controls are effective for mitigation only if they are carried out at the time of maximum exposure rather than at the time of detection of infection. The primary challenge is to develop quantitative relations between vector abundance and other observables that can be used to identify such peaks in advance with sufficient accuracy.

Since malaria, as a mosquito-borne disease is strongly modulated by weather, quantitative relations between the vector abundance and weather variables can enable identification of peaks of vector population through meteorological monitoring and forecast. The life cycle of the malaria vector is a complex function of weather variables like temperature, rainfall and humidity. Temperature affects the developmental period related to different stages of a mosquito’s lifecycle: blood feeding rate, gonotrophic cycle [physiological process consisting of digestion of blood-meal and development of ovaries], and longevity [5]–[8]. However, the dependence of mosquito vector on meteorological variables can be quite complex, and other environmental variables like land use and land cover also affect vector dynamics [9]–[11]. In particular, local environmental characteristics, such as altitude, climate and land use, can significantly impact on phenology and population dynamics of mosquito larvae. It has been shown that rainfall significantly affects larval mosquitoes by flushing them out of their aquatic habitat and thus killing them [7]. Experiments with simulated dry conditions show rainfall and dryness to have significant and complex effects on larval survival [8]. However, these effects also depend on the species of the vector. Study conducted at different altitudes in the Lake Victoria basin, showed [12] significant differences in larval abundance depending on weekly rainfall intensity. However, the genesis and the survival of mosquitoes depend on a combination of meteorological variables within specific ranges. Thus vulnerability to malaria due to climate change cannot be assessed from the trend of a single meteorological variable. Quantitative, validated models of malaria epidemiology based on a combination of all the relevant weather variables are therefore critical for such assessment.

The usefulness of mathematical (dynamical as well as static) models to gain insight into the epidemiology of malaria has been recognized early [13]–[16] as well as in recent times [17]. The static models are basically those that rely on statistical relations based on past observations or static relations between various variables under given conditions. In the dynamic models, the system evolves through time variations of the variables that govern the epidemic.

Mathematical models of mosquito-borne pathogen transmission were developed quite early [13]. More complex deterministic models, suitable for a large community and also stochastic model relevant to small populations in which infections reach very low finite numbers, were considered in the sixties with the advent of computing facilities [14]. Since then several workers have contributed to the development of the Ross- Macdonald model. These Ross-Macdonald models are best defined by a consensus set of assumptions. The Ross-Macdonald theory has since played a central role in development of research on mosquito-borne pathogen transmission and the development of strategies for mosquito-borne disease prevention[15]–[17]. There have also been several recent attempts to develop more comprehensive dynamical malaria models [18]–[20]. The Liverpool Malaria Model (LMM), a mathematical-biological model of malaria parasite dynamics uses daily temperature and precipitation data; the mathematical formulation considers key processes related to the growth and size of the vector population [20]. However, the model as yet does not consider humidity explicitly. Statistical methods, like Autoregressive Integrated Moving Average (ARIMA) analysis, have been also applied to assess relationship between environmental variables like land use and incidence of *Plasmodium falciparum* infection [21].

Many of these models are quite comprehensive in relating entomological parameters to malaria transmission and include many parameters like age-at-infection, human blood index, entomological inclusion rate, vectorial capacity etc [22]–[23] but often do not involve all the weather variables explicitly. Quantities like vectorial capacity, a measure of transmission risk [24] **,** may involve climate variables but only indirectly and are generally limited to entomological parameters such as the duration of extrinsic incubation [25]–[26]. In particular, rarely all the three meteorological parameters have been included and related to observed epidemiology.

There are models of population dynamics of vector that consider effects of variables like temperature and rainfall [11], but without validation against epidemiology. The incubation period for malaria parasites within the mosquito is extremely sensitive to temperature. However, many models are based on mean monthly temperatures, and thus ignore significant variation in temperatures at daily and diurnal scales [27]–[29]. Further, a model of epidemiology needs validation against observations for a given location [30]. The dependence of vector population on environmental parameters can be highly location–specific. While temperature, rainfall, humidity and other variables like wind and duration of day light are all known to be important for mosquito life cycle, the relative roles of these variables may depend on a given location. For example, the periodicity and the amplitude of mosquito population [abundance peaks] in northern Australia were found to strongly depend on frequency and the amplitude of tides [31]; a land-locked region, on the other hand, requires different considerations [32].

## Methods

### Ethics Statement

We declare that the data on epidemiology in this study was collected and compiled by the co-authors from CSIR-IICT based on records at the Public Health centres in Arunachal Pradesh and was analyzed anonymously; no particular patient by name was involved.

### Study Area

Situated between latitude 26 30′ N and 29 30′ N and longitude 91 30′ E and 97 30′ E, Arunachal Pradesh is dominated by the Himalayan system and different attitudinal variations. It stretches from snow-capped mountains in the north to the plains of the Brahmaputra valley in the south. The climate is warm and humid at the lower altitudes, with cold climate in the higher altitudes. The valleys are covered by swampy dense forest; forested terrain and perennial streams are congenial for rapid multiplication and longevity of malaria vectors. Agriculture is the primary driver of the economy; nearly 80% of the population is engaged in agriculture. The 12 districts of our study area, Arunachal Pradesh, are characterized by different elevations (Table 1) and are all endemic to malaria. These districts also exhibit large variations in annual mean values of meteorological variables (Table 2); the mean annual maximum and minimum temperatures, as well as daily humidity and rainfall show that the districts can easily move in and out of genesis range due to fluctuations in these variables at monthly and daily scales.

**Table 1:** The twelve districts of Arunachal Pradesh with elevation, average human population and number of public health centers (PHCs).

Districs	Elevation(Mts)	Population(N_H_)	PHCs
Tirap	1278	95262	6
Changlang	580	85146	11
Lohith	750	94711	10
Lower Dibang Valley	2655	53419	8
East Siang	455	77469	17
West Siang	750	101445	13
Upper Siang	1500	27735	6
Upper Subansiri	1500	39309	7
Papum Pare	355	133652	10
Kurung Kumey	2450	38697	8
East Kameng	780	22807	8
West Kameng	810	44329	7

The twelve districts of Arunachal Pradesh with elevation, average human population and number of public health centers (PHCs).

### Epidemiological Data

The data was collected from the Directorate of Health, Govt. of Arunachal Pradesh, which consists of epidemiological aspects of Malaria cases from Primary Health Centres (PHCs) belonging to 12 districts of Arunachal Pradesh. The data comprised of number of blood samples collected (N_BSC_), number of blood samples that tested positive for either *Plasmodium vivax* (N_PV_) or *Plasmodium falciparum (N*_PF_) infection, or mixed infection; thus blood sample positive (N_BSP_) is the sum total of N_PV_, N_PF_ and mixed type. The data for 2006–2010 were collected at a monthly scale; this monthly data was augmented by records of annual data for the period of 2002–2005.

The epidemiological data were collected by using both active and passive surveillance methods. Samples with fever or with history of fever over past few days (clinical cases)/without fever were screened for malaria parasite by finger prick blood-smear using standard microscopic technique. Both thick and thin blood-smears stained with Jaswant Singh Bhattacharya stain were examined by using microscope for malaria parasite and species identification before declaring the slide negative/positive. The positive cases were then treated with antimalarial drugs according to the type of parasite species following the World Health Organization (WHO) recommendations.

The blood smears were collected from all the inhabitants of the area thus covering not only passive data but also active surveillance data, and of real toll cases will not exist. As all the people are covered in the selected area, asymptotic cases are essentially ruled out and biases among people do not arise; even if they cannot visit the health centers, the health volunteers reach the respective individual and follow up the malaria treatment.

### Thresholds for Vector Genesis

The aquatic stages of *Anopheline* mosquitoes in the Tropics do not develop or breed below 16°C, and the minimum temperature for *Plasmodium falciparum* (PF) malaria parasite development is experimentally between 16°C and 19°C and varies among mosquito species. The minimum temperature required for development of *Plasmodium vivax* (PV) parasite in *anopheline* mosquitoes ranges from 14.5–16.5°C. While temperature, rainfall, and humidity play critical roles, other variables such as wind and the duration of daylight are also important. These factors are also important in the survival and transmission rate of mosquito borne pathogens; in particular, temperature affects their rate of multiplication in the insect [17]. On the other hand transmission can not occur if the development time of the pathogen exceeds the life span of the insect [31]. If water temperature rises, the larvae take a shorter time to mature and consequently there is a greater capacity to produce more offspring during the transmission period [4].The threshold for vector genesis and survival were adopted (Table 2) from the values available in the literature.

**Table 2:** Meteorological variables with observed minimum and maximum daily values.

Districts	Temperature(°C)		Humidity (%)		Rainfall(mm)	
	Minimum	Maximum	Minimum	Maximum	Minimum	Maximum
**Genesis Threshold**	**18(T_min_)**	**32(T_max_)**	**20(Q_min_)**	**90 (Q_max_)**	**1(R_min_)**	**20(R_max_)**
Tirap	8	27	25	85	2	20.5
Changlang	10	30	18	82	2.5	22.6
Lohit	11	32	16	75	1.3	30.4
L/D Valley	6	34	23	88	1.7	18.5
East Siang	13	28	21	86	2.4	40.4
West Siang	15	35	17	80	1	26
Upper Siang	6	26	30	91	1.1	22.5
U/Subansiri	8	29	26	89	1.4	19.3
Papum Pare	7	30	28	85	1.6	22.1
K/Kumey	10	29	19	81	1.9	27.8
E/Kameng	16	29	18	84	2.1	32.6
W/Kameng	14	32	25	87	1.4	19.3

Meteorological variables used in the model for genesis [24] and observed minimum and maximum daily values in a year during (2000–2010) in each of the twelve districts. The basic meteorological data is from global NCEP Reanalysis data averaged for the respective district. The first row of values signifies thresholds adopted for mosquito genesis and survival [24], [29].

### Meteorological Data

The daily values of near surface temperature and near surface humidity were obtained from global NCEP (National Centre of Environmental prediction) reanalysis data in a 2.5°*2.5° grid over the respective district. The 24-hour accumulated rainfall was obtained from global NCEP Reanalysis data in a 1.8°*1.8° grid over the respective district. The daily data at district level was created through interpolation*;* the interpolated NCEP Reanalysis has been shown to have good correspondence with high resolution data from other sources over the region [33]. The advantage of NCEP Reanalysis is that it provides a comprehensive and consistent long-period data for all the three variables on a grid.

## The Model

### Genesis

Our model of malaria epidemiology is based on vector population as a function of weather variables; exposure and transmission leading to malaria infection are considered functions of number of human hosts as well as vectors. The vector population is assumed to be governed by three weather variables, with the number of vectors determined by
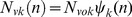
(1)


Where N_vk_(n) is the vector population on day n for the location (district) k, and N_vok_ is a constant [Genesis Potential].The quantity Ψ_k_ [n] is 1 if the day n is a vector genesis day, and zero otherwise; a day is counted as one for vector genesis if
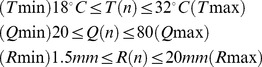
(2)


The variables Ximin and Ximax represent, respectively, minimum and the maximum values of the variable Xi beyond which mosquito genesis does not take place. The values of Tmin, Tmax, Rmin, Rmax, Qmin and Qmax for temperature, humidity and rainfall (Table 2) have been adopted from standard data set [31], [6], [22], [29]. The condition of genesis for each day and for each meteorological variable is then evaluated over each district from daily mean values using data from NCEP Reanalysis [32]. In our scenario presented by equations (1) and (2), vector abundance is a regenerative process based on daily meteorological conditions. In principle, there will be also residual vectors from the previous days (up to a lag of typical life span of a vector); for simplicity, we absorb this factor in calibrating the scaling factor, as discussed subsequently.

For the combined case, each of the three variables T(n), Q(n) and R(n) must satisfy the genesis condition for Ψ_k_(n) to be equal to 1. Monthly values of vector population, N_V_(m), are then calculated by summing over 30 days for each month:
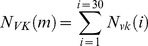
(3)


Where N_VK_ (m) is the vector population for month m and district k.

### Exposure and Transmission

We next relate the entomological scenario represented by equations (1)–(3) to epidemiology by prescribing exposure and transmission. Infection depends both on the number of bites (exposure) and the transmission of the parasite. The exposure depends on the encounters of mosquito and human, which is a complex function of migration and movement of the human population as well as the vector population. Only a fraction of the total mosquito population will eventually bite the host (human); however, it is reasonable to assume that a larger vector density leads to enhanced exposure and thus more bites. The number of exposures or bites (N_E_) thus depends on both the number of hosts available and the size of the vector population, and is assumed to be proportional to the total human population (N_H_) and the number of vectors (N_V_); thus
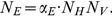
(4)


Where α_E_ is a constant (coefficient of exposure) of proportionality. Not all bites result in an infection. Only a fraction of the mosquitoes will carry either of the parasites *Plasmodium falciparum* (PF) or *Plasmodium vivax* (PV) to infect human host. The actual cases of malaria thus depend on transmission, which may be considered primarily a function of the fraction of mosquitoes that carry either PV or PF, as well as the immunological history of the population. We thus consider the actual number of malaria case (N_M_) proportional to the number of exposures with a transmission coefficient α_T_ (<1):
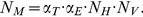
(5)


Equation (5) is then applied to each of the 12 districts, with α_T_, α_E_, N_H_ and N_V_ characteristic of a given district (k). Equations (1) to (5) are then fed into a computer program along with the daily meteorological data to simulate genesis at daily scale for each district.

To obtain estimates of α_T_ and α_E_ we first estimate the exposure coefficient (α_E_) and the transmission coefficient (α_T_) in an objective manner, based on the observed parameters as follows:
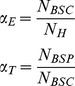
(6)


These estimates are then used for calibration of the model.

While both α_E_ and α_T_ vary from district to district and from month to month for a given district (Table S1 (a-l)) in a wide range, the variation for α_T_ with month is confined to a small range, implying a nearly uniform coefficient of transmission for a given district, as expected. As transmission is considered to depend only on the immunological history, we assume a single value for it for a district for the whole year. This value, computed based on average of monthly data, is then kept constant for all the years for a given district.

The extent and duration of outdoor human activities tend to change with the season, leading to changes in the number of potential encounters between mosquito and human; this results in changes in the exposure coefficient, as expected. We have therefore considered an exposure coefficient α_E_ for each month and for each district (Table 3). The values of α_E_ adopted were estimated first for each district for five years (2006–2010) based on data on N_H_, N_BSC_ and N_BSP_ (Table S1 (a-l)); average values (Table 3) were then adopted to simulate epidemiology (N_M_). It may be seen that α_E_ for each district shows a distinct annual cycle, with low values in the winter and significantly larger values in the summer (Table 3); this is consistent with more human outings for foraging, agriculture etc in the summer months.

**Table 3:** Coefficient of exposure for 12 months.

Month	Districts in Arunachal Pradesh											
	Tirap	Changl-ang	Lohit	LowerDibangValley	EastSiang	WestSiang	UpperSiang	UpperSub-ansiri	PapumPare	KurungKumey	EastKameng	WestKameng
Jan	1.0	1.4	0.9	1.7	3.6	1.0	1.4	1.0	2.5	0.9	1.9	1.4
Feb	1.0	1.0	1.1	1.8	4.0	1.2	1.3	2.5	1.8	0.7	1.5	1.4
Mar	1.1	1.5	1.5	2.2	4.7	1.9	1.2	1.4	2.9	0.8	2.2	1.4
Apr	1.0	1.4	1.9	2.1	5.9	1.9	1.0	1.2	5.1	1.3	4.6	1.1
May	1.7	2.4	4.2	4.6	9.3	3.8	1.2	1.3	6.1	1.3	8.5	1.2
Jun	4.0	6.1	9.0	15.5	9.7	6.0	1.6	2.6	7.3	1.4	8.9	1.3
Jul	3.3	6.8	13.1	14.9	23.4	7.4	2.2	4.8	9.3	1.5	9.7	1.3
Aug	3.0	5.0	8.0	5.9	19.5	5.5	2.1	2.6	5.9	1.5	21.0	1.5
Sep	2.5	5.8	7.0	6.9	11.6	4.6	2.0	3.4	4.8	1.7	19.4	1.0
Oct	1.9	7.2	5.7	6.3	10.1	2.3	1.3	2.8	6.3	2.0	6.6	1.4
Nov	1.7	8.8	5.0	4.3	7.8	1.6	1.0	1.7	3.9	1.3	13.9	1.0
Dec	1.0	4.5	2.1	3.4	3.9	1.3	0.9	1.1	1.6	0.9	2.6	1.4
**Avg α_E_**	1.9	4.3	5.0	5.8	9.5	3.2	1.4	2.2	4.8	1.3	8.4	1.3
**Avg α_T_**	0.1	0.1	0.1	0.1	0.2	0.2	0.1	0.2	0.1	0.2	0.3	0.1

The twelve districts of Arunachal Pradesh with the coefficient of exposure (α_E_ *10–3) for each month. The last row represents the average transmission coefficient α_T_ (N_BSP_/N_BSC_).

### Calibration of the Model Parameters

It should be noted that it is not necessary to substitute the parameters α_T_ and α_E_ in equation (4) separately. Because of the multiplicative nature of the coefficients in equation (4) the computed value of N_M_ depends on a single constant estimated by using α_T,_ α_E_ and N_vok_. In principle, the calibration of the single parameter is independent of these estimates, and use of α_T,_ α_E_ and N_vok_ in the process is essentially for our understanding and interpretation. We therefore determine a single parameter, guided by the estimates of α_T_ and α_E,_ that provides best fit (minimum average absolute error) between observed and simulated epidemiology. Here observed data refers to epidemiology in terms of blood samples that tested positive (N_BSP_).

The final value of N_vok_ for a district is then calculated assuming a constant genesis potential of N_vok_ = 100000, and multiplying it by α_T_ [with no year to year variability for a district] and α_E_ (month-dependent but no year to year variability).The value of N_vok_ was kept identical for all the 12 districts and for all the years; thus the variability in simulated epidemiology is a result of variation in vector genesis days and exposure coefficient at monthly scales; at interannual scale the variability in simulated epidemiology is due to interannual variations in the vector genesis days.

The calibration is then carried out as follows:

For each district (k), the error in simulated epidemiology is calculated for each month(m)as:
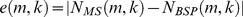



Where N_M_ is given by equation (5), and N_MS_ refers to the simulated value.

To avoid use of in-sample data, so that our model is close to a forecast model, we have used α_T_ as an average value; α_E_ is estimated from N_BSC_ which is correlated to but separate from the epidemiological data (N_BSC_).

### Validation

Since the year to year variability in α_E_ and α_T_ have been eliminated, the inter annual variability in the simulated epidemiology results from the meteorological drivers. In general, it is desirable to calibrate these parameters on an independent data set to evaluate forecast skill. However, the size of the total sample (5 for a given month and district) makes such a partition impractical. However, we have used additional years (2006–2010) to compute annual epidemiological load using the(independently) calibrated model. Still, the current model provides estimate of potential forecast skill rather than that of actual forecast.

It is of course possible, and likely, that α_E_ calculated in the above manner is underestimated due to unreported asymptomatic cases. However, such an underestimation in our case only changes the scaling factor due to multiplicative nature of the formulation (equation 5). For the same reason, underestimation of the number of bites does not affect our formulation, although it affects our scaling. The model is then applied to the study area, with N_BSP_ representing the number of epidemiological cases (N_M_).

## Results

### Spatio-temporal Variability of Malaria Over Arunachal Pradesh

**Figure 1:**
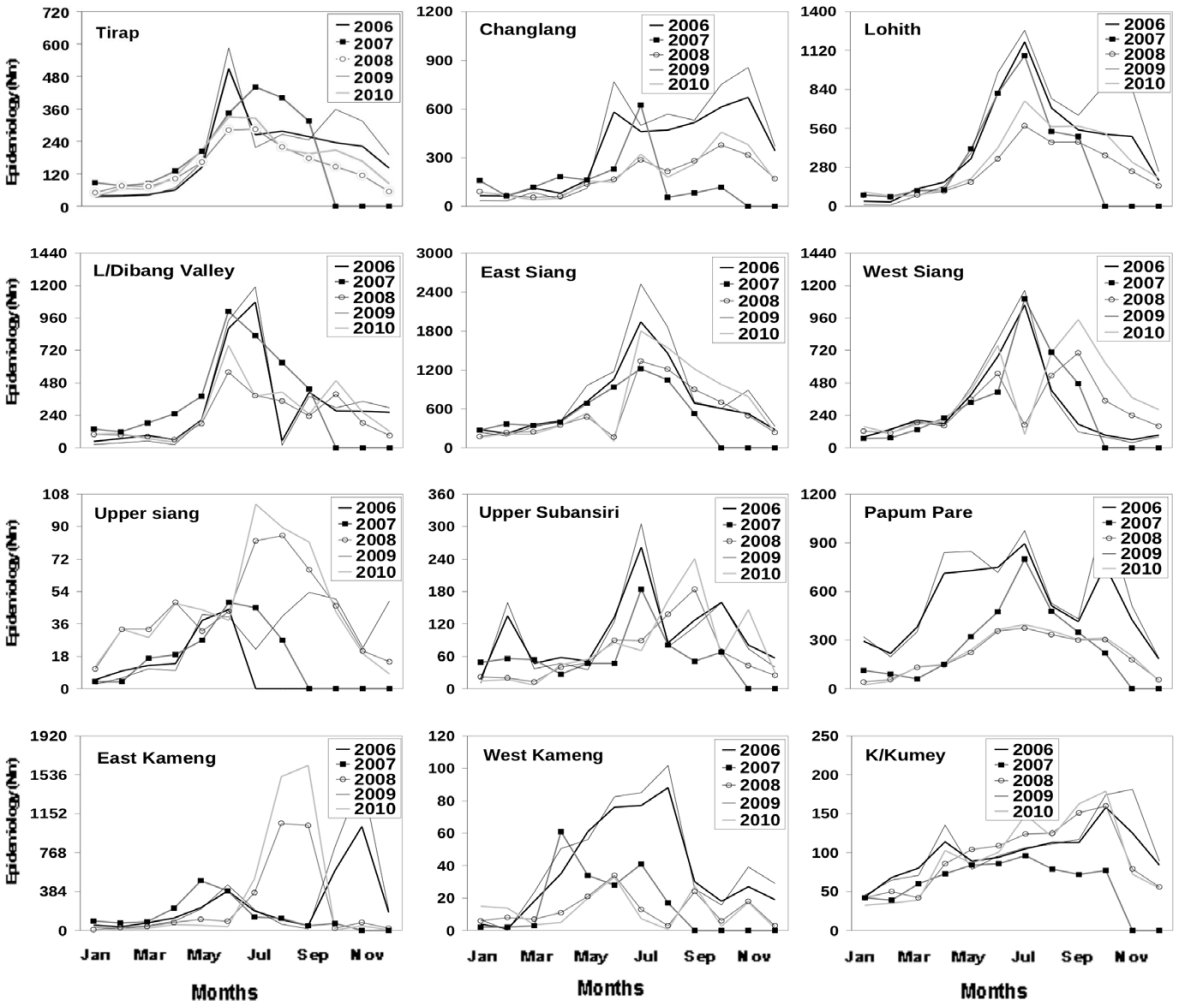
Inter-annual variability in epidemiology over Arunachal Pradesh. Inter-annual variability in epidemiology of Blood Sample Positive (N_BSP_).For the years 2006 (thick line), 2007 (thin line with square point), 2008 (light line with circle point), 2009 (thin line) and 2010 (light line) for the twelve districts in Arunachal Pradesh.

The twelve districts (Table 1) exhibit significant differences in the amplitude as well as the structure of the annual cycle of epidemiology (N_BSP_, Figure 1).Although the peak occurs generally in the monsoon months (June-September) there are appreciable year-to-year variability (Figure 1) for any given district. Further, the 12 districts follow independent patterns of annual cycle and inter annual variability of epidemiology; the results for one district can not be used to infer epidemiology of another. These 12 districts represent a wide range in terms of annual mean values of temperature, humidity and rainfall (Table 2). A major challenge for a model of malaria over this region is thus to successfully capture the annual cycle and the inter annual variability of epidemiology for each district.

### Relative Roles of the Weather Variables in Malaria

**Figure 2:**
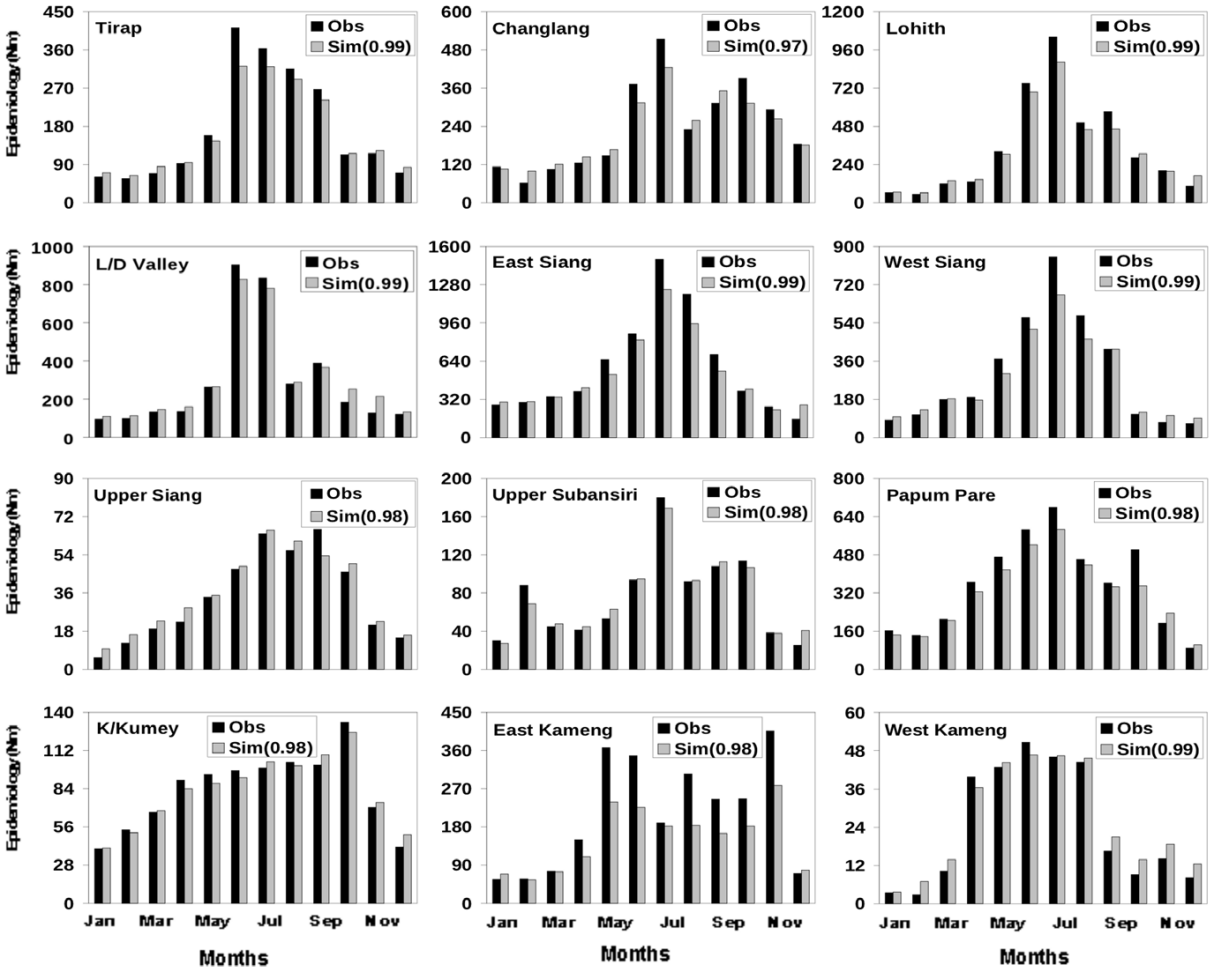
Monthly climatology of observed and simulated epidemiology for the twelve districts. Monthly climatology (2006–2010) of observed (N_BSP_) and simulated (N_M_) epidemiology for the twelve districts calculated using daily temperature, surface humidity and 24 hour accumulated rainfall. The meteorological parameters (temperature humidity and rainfall) for each district have been adopted for the corresponding year from NCEP daily reanalysis data. The number in the bracket represents the correlation coefficient between observed and simulated epidemiology for the respective district.

All the three weather variables: temperature, rainfall and humidity, are known to play important roles in vector genesis. Nonetheless, simulation of epidemiology using equation (1)–(5) was first carried out with each of the three meteorological variables separately to examine and quantify their relative roles as well as to understand the minimum complexity required in the model. An analysis of simulations with only temperature (Figure S1), only humidity (Figure S2) and only rainfall (Figure S3), however, showed large errors. It was also found that the number of days that allow vector genesis varied appreciably (was considerably reduced) when all the three weather variables were considered as against a single weather variable, as expected. The best results were obtained with all the three weather variables (Figure 2).

### Comparison of Observed and Simulated Monthly and Annual Climatology of Malaria

**Figure 3:**
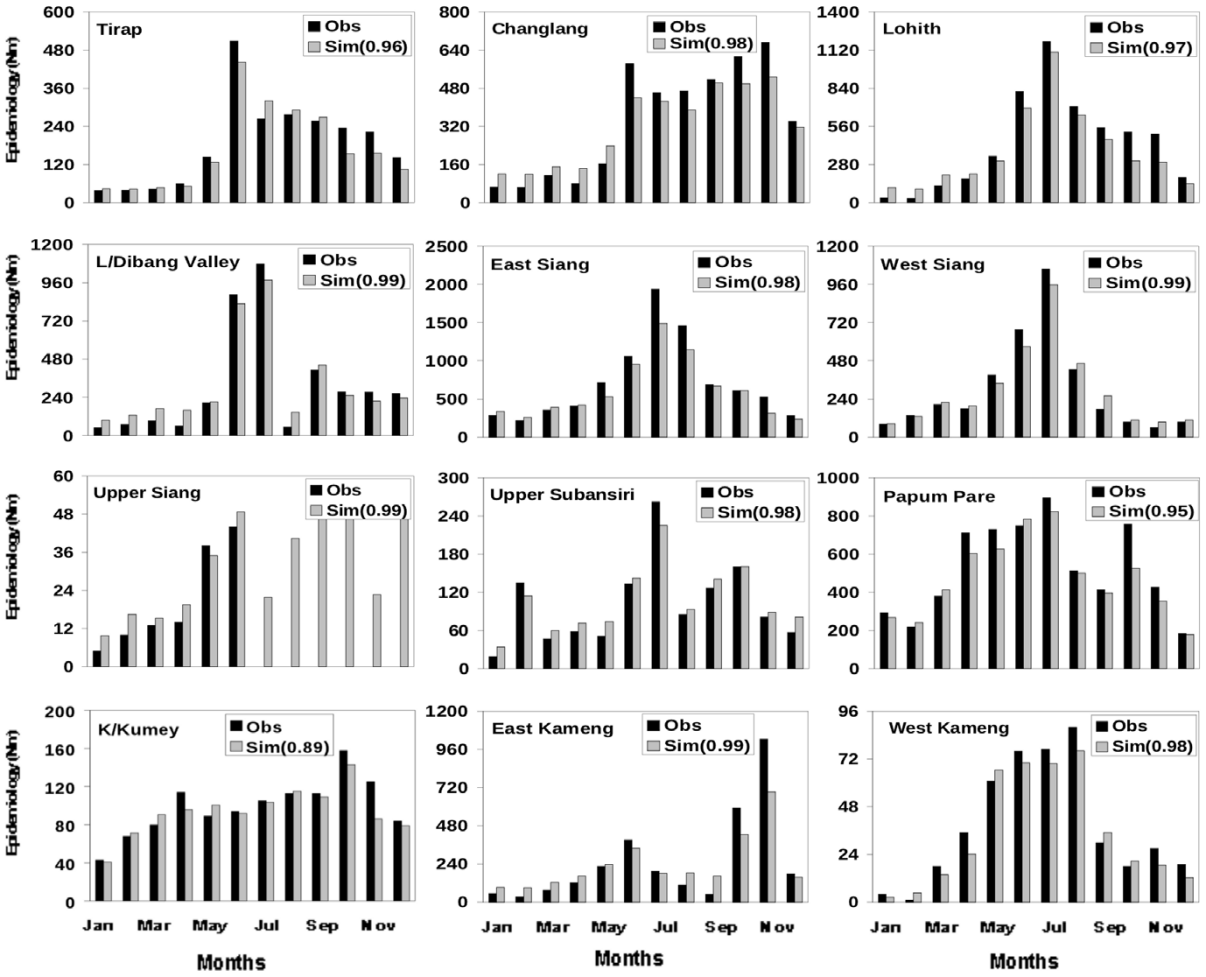
Annual cycle of observed and simulated malaria epidemiology: 2006. Annual cycle of observed (N_BSP_) and simulated (N_M_) malaria epidemiology based on calculation of N_M_ using daily temperature, surface humidity and 24 hour accumulated rainfall for the twelve districts for the year of 2006. The meteorological parameters (temperature, humidity and rainfall) for each district have been adopted for the corresponding year form NCEP reanalysis data. The number in the bracket represents the correlation coefficient between observed and simulated epidemiology for the respective district.

**Figure 4:**
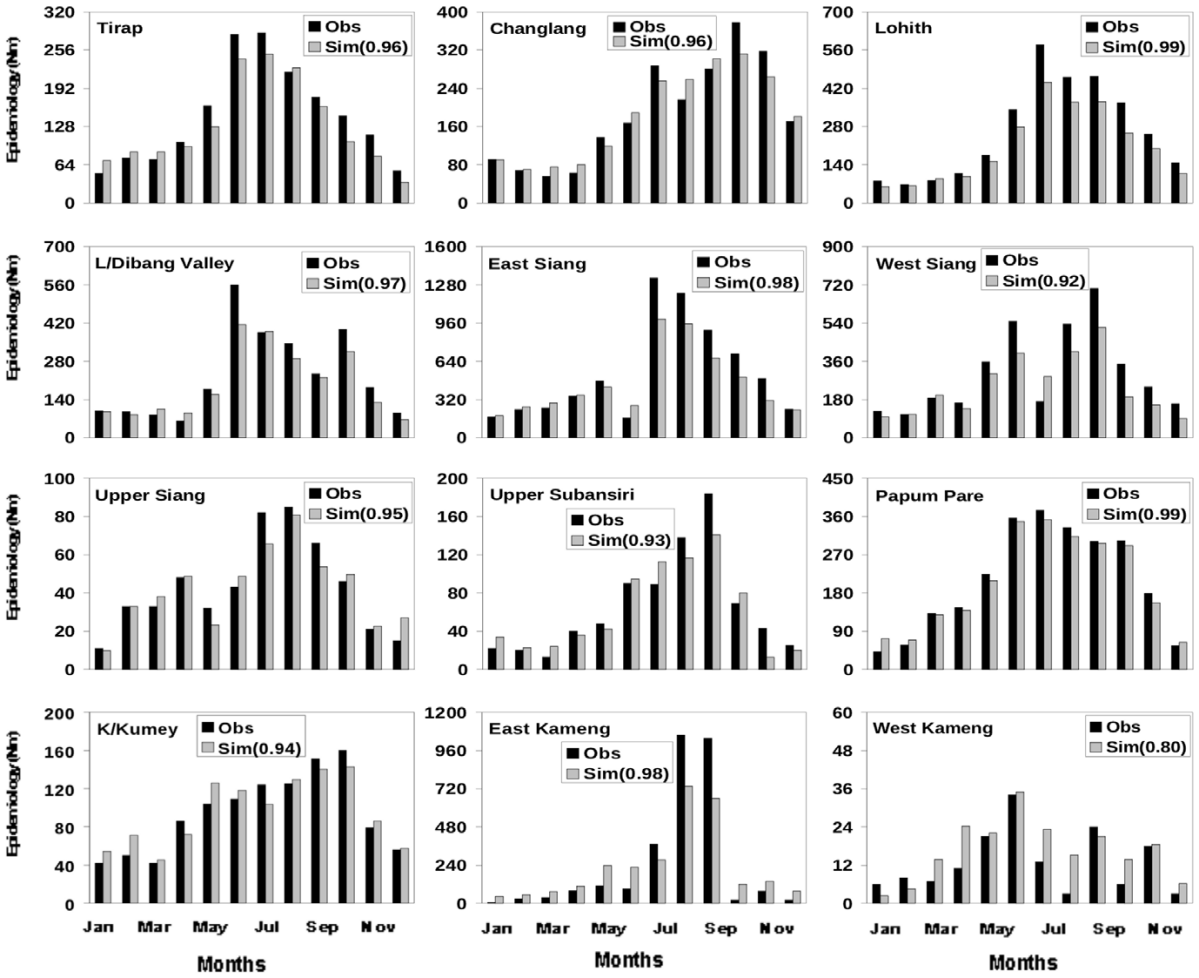
Annual cycle of observed and simulated malaria epidemiology: 2008. Annual cycle of observed (N_BSP_) and simulated (N_M_) malaria epidemiology based on calculation of N_M_ using daily temperature, surface humidity and 24 hour accumulated rainfall for the twelve districts for the year of 2008. The meteorological parameters (temperature, humidity and rainfall) for each district have been adopted for the corresponding year form NCEP reanalysis data. The number in the bracket represents the correlation coefficient between observed and simulated epidemiology for the respective district.

A comparison of monthly climatology (over 2006–2010) of N_BSP_ from observation and N_M_ from simulations, with vector abundance derived from simulations with all the three weather variables, shows excellent agreement (Figure 2) for most of the twelve districts. The simulations match the observed (multiple) peaks (Figure 2) in most districts. For example, while the model shows a gradual decline of malaria cases beyond June for Tirap in accordance with the observations [Figure 2, top panel], it shows a sharp, isolated peak in August-September for East Kameng, and the multiple peaks for districts like Upper Siang and Kurung Kumey. In terms of individual year also the simulations match the observations well. A comparison of simulated and observed epidemiology for the 12 districts for 2006 (Figure 3) and 2008 (Figure 4) emphasizes the appreciable year to year variability in essentially all the 12 districts. For each of these two years the simulated epidemiology compares well with the corresponding observation, with significant (99%) correlation between the two; a similar conclusion holds for the other years (Figure S4–S6). The correlation coefficient between the monthly values of observed and simulated epidemiology for each district**,** as given in the respective figure, is significant above 99% confidence level for the degrees of freedom involved.

### Annual Epidemiology Load from Simulation and Observation

**Figure 5:**
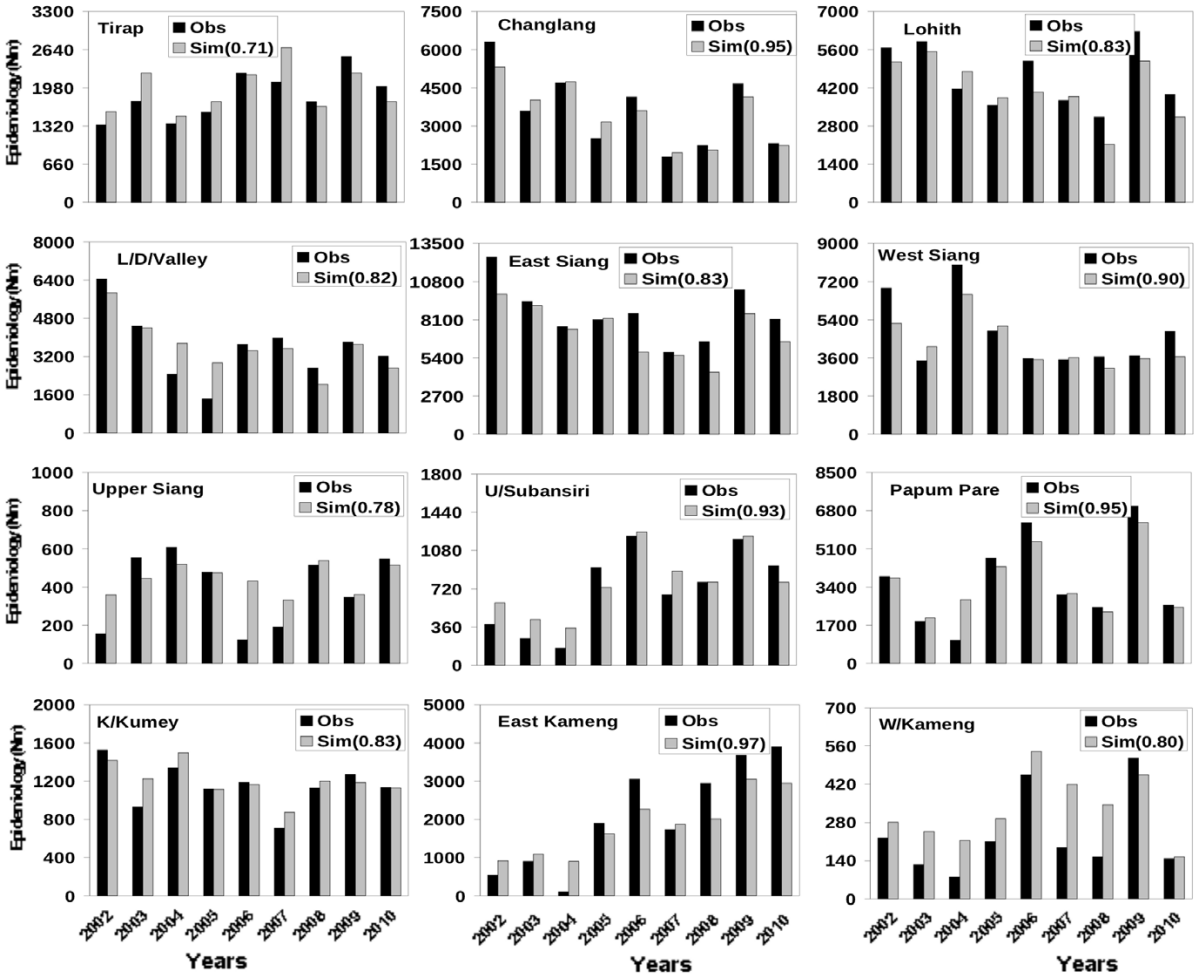
Inter annual variability in observed and simulated malaria epidemiology. Inter annual variability in observed (N_BSP_) and simulated (N_M_) malaria epidemiology for the twelve districts calculated using daily temperature, surface humidity and 24 hour accumulated rainfall. The meteorological parameters (temperature, humidity and rainfall) for each district have been adopted for the corresponding year from NCEP daily reanalysis data. The number in the bracket represents the correlation coefficient between observed and simulated epidemiology for the respective district.

While data for epidemiology at monthly scale were available for 2006–2010, annually averaged data was available for the period (2002–2005). Together with the annually averaged data for 2006–2010, we have examined the interannual variability in the simulations using the same values of α_T_ and α_E_ that were used for 2006–2010. The simulated interannual variability agrees well with the observed variability for all the 12 districts (Figure 5). While most of the districts do not show any noticeable trends, a few do show increasing (like East Kameng and Upper Subansiri) and decreasing (like Changlang) trends, which are well captured by the simulations.

## Discussion and Conclusions

While vulnerability to malaria in a changing climate is a growing concern [34], reliable regional projections require validated relationship between the weather variables and epidemiology; our model provides such relations over a region highly endemic but little explored. It is shown that the observed annual cycles (2006–2010) and inter annual variability (2002–2010) of epidemiology are well simulated for each of the 12 districts. While no single weather variable like temperature can reproduce the observed epidemiology, a combination of temperature, rainfall and humidity in the model provides an accurate description of the annual cycle as well as inter annual variability over all the 12 districts. The model can be used at short scales for outbreak forecast through high resolution weather monitoring. While the validation has been carried out over a specific region, the basic algorithm is easily applicable to other regions. For a given region, the parameters for exposure and transmission can be recalibrated periodically, based on primary data on population. However, validation with longer and independent epidemiological data is required for more robust estimation of realizable skill.

Regionally, climate change can increase as well as reduce vulnerability as the local climate shifts in or out of the genesis regime; because of the use of genesis days based on three weather variables, the model can be used to assess such shifting vulnerability due to regional climate change in a comprehensive manner. However, transmission also depends on the life cycle of the malaria parasite inside the mosquito and the human host. Reduction in the duration of gonotrophic cycle and in the extrinsic incubation period of malaria parasite is related to increased rate of transmission; these effects may become important in extreme cases of climate change.

There exist several avenues for improvement. Evidence suggests that certain malaria vectors can spend large parts of their adult life resting indoors. Differences in indoor vs outdoor environments can lead to large differences in the limits and the intensity of malaria transmission. There is thus need to understand and model relationship between mosquito resting behavior and the associated micro climate, and to broaden assessments of transmission ecology and risk to consider the potentially important role of endophily [35].

The development rate of parasites and pathogens within vectors typically increases with temperature. Many current disease models ignore the interactive effects of environmental temperature on multiple host-disease life-history traits; there is need to understand this complexity and incorporate in the models [36]. In particular, there is need for including variability of the meteorological variables at shorter time scales [37].

It is clear that effective implementation of identification and eradication of malaria risk requires fine scale stratification of the epidemiological situation [38]. Analysis of risk heterogeneity at the household scale by Geographical Information System (GIS) methods can lead to target preventive actions more accurately on the high-risk zones identified. However, this needs to be carried out in a hierarchical multi-scale environment, starting from identification of macro endemic zone for surveillance to final implementation at house-hold scale. It is unlikely that meteorological monitoring at house hold level with sufficient geographical coverage will be available in near future. Our model provides a means for such surveillance at district level.

It is quite possible that some of the errors in the model simulations originate from different relations between vector and meteorological factors over locations. However, we would like to treat this as a second order effect. In particular, a location (variety-dependent) range of meteorological variables can be incorporated once these are identified with sufficient precision.

## Supporting Information

Figure S1 **Monthly climatology of observed and simulated epidemiology: Only temperature.** Monthly climatology (2006–2010) of observed (N_BSP_) and simulated (N_M_) epidemiology for the twelve districts calculated using only daily temperature. The meteorological parameter (temperature) for each district has been adopted for the corresponding year from NCEP daily reanalysis data. The number in the bracket represents the correlation coefficient between observed and simulated epidemiology for the respective district.
(DOC)

Figure S2 **Monthly climatology of observed and simulated epidemiology: Only humidity.** Monthly climatology (2006–2010) of observed (N_BSP_) and simulated (N_M_) epidemiology for the twelve districts calculated using only surface humidity. The meteorological parameter (humidity) for each district has been adopted for the corresponding year from NCEP daily reanalysis data. The number in the bracket represents the correlation coefficient between observed and simulated epidemiology for the respective district.
(DOC)

Figure S3 **Monthly climatology of observed and simulated epidemiology: Only rainfall.** Monthly climatology (2006–2010) of observed (N_BSP_) and simulated (N_M_) epidemiology for the twelve districts calculated using only 24 hour accumulated rainfall. The meteorological parameter (rainfall) for each district has been adopted for the corresponding year from NCEP daily reanalysis data. The number in the bracket represents the correlation coefficient between observed and simulated epidemiology for the respective district.
(DOC)

Figure S4 **Annual cycle of observed and simulated epidemiology for the year 2007.** Annual cycle of observed (N_BSP_) and simulated (N_M_) malaria epidemiology based on calculation of N_M_ using daily temperature, surface humidity and 24 hour accumulated rainfall for the twelve districts for the year of 2007. The meteorological parameters (temperature, humidity and rainfall) for each district have been adopted for the corresponding year form NCEP reanalysis data. The number in the bracket represents the correlation coefficient between observed and simulated epidemiology for the respective district.
(DOC)

Figure S5 **Annual cycle of observed and simulated epidemiology for the year 2009.** Annual cycle of observed (N_BSP_) and simulated (N_M_) malaria epidemiology based on calculation of N_M_ using daily temperature, surface humidity and 24 hour accumulated rainfall for the twelve districts for the year of 2009. The meteorological parameters (temperature, humidity and rainfall) for each district have been adopted for the corresponding year form NCEP reanalysis data. The number in the bracket represents the correlation coefficient between observed and simulated epidemiology for the respective district.
(DOC)

Figure S6 **Annual cycle of observed and simulated epidemiology for the year 2010.** Annual cycle of observed (N_BSP_) and simulated (N_M_) malaria epidemiology based on calculation of N_M_ using daily temperature, surface humidity and 24 hour accumulated rainfall for the twelve districts for the year of 2010. The meteorological parameters (temperature, humidity and rainfall) for each district have been adopted for the corresponding year form NCEP reanalysis data. The number in the bracket represents the correlation coefficient between observed and simulated epidemiology for the respective district.
(DOC)

Table S1 Coefficients of transmission and exposure for the 12 months.
(DOC)
