# Supplementary material for: A Model of Malaria Epidemiology Involving Weather, Exposure and Transmission Applied to North East India
Source: PLoS One. 2012 Nov 27;7(11):e49713. doi: 10.1371/journal.pone.0049713 (PMC3507888; doi:10.1371/journal.pone.0049713)
Supplement: Figure S1 — Monthly climatology of observed and simulated epidemiology: Only temperature. Monthly climatology (2006–2010) of observed (NBSP) and simulated (NM) epidemiology for the twelve districts calculated using only daily temperature. The meteorological parameter (temperature) for each district has been adopted for the corresponding year from NCEP daily reanalysis data. The number in the bracket represents the correlation coefficient between observed and simulated epidemiology for the respective district. (DOC) [file pone.0049713.s001.doc]

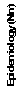

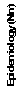

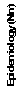

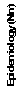

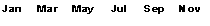

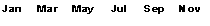

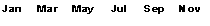

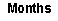

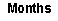

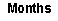


**Figure S1:** Monthly climatology (2006-2010) of observed (N_BSP_) and simulated (N_M_) epidemiology for the twelve districts calculated using only daily temperature. The meteorological parameter (temperature) for each district has been adopted for the corresponding year from NCEP daily reanalysis data. The number in the bracket represents the correlation coefficient between observed and simulated epidemiology for the respective district.
