# Supplementary material for: A Model of Malaria Epidemiology Involving Weather, Exposure and Transmission Applied to North East India
Source: PLoS One. 2012 Nov 27;7(11):e49713. doi: 10.1371/journal.pone.0049713 (PMC3507888; doi:10.1371/journal.pone.0049713)

**Figure S5:** Annual cycle of observed (N_BSP_) and simulated (N_M_) malaria epidemiology based on calculation of N_M_ using daily temperature, surface humidity and 24 hour accumulated rainfall for the twelve districts for the year of 2009. The meteorological parameters (temperature, humidity and rainfall) for each district have been adopted for the corresponding year form NCEP reanalysis data. The number in the bracket represents the correlation coefficient between observed and simulated epidemiology for the respective district.


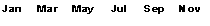

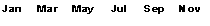

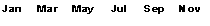

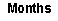

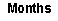

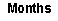

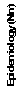

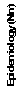

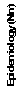

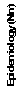

Supplement: Figure S5 — Annual cycle of observed and simulated epidemiology for the year 2009. Annual cycle of observed (NBSP) and simulated (NM) malaria epidemiology based on calculation of NM using daily temperature, surface humidity and 24 hour accumulated rainfall for the twelve districts for the year of 2009. The meteorological parameters (temperature, humidity and rainfall) for each district have been adopted for the corresponding year form NCEP reanalysis data. The number in the bracket represents the correlation coefficient between observed and simulated epidemiology for the respective district. (DOC) [file pone.0049713.s005.doc]
