# Supplementary material for: A Model of Malaria Epidemiology Involving Weather, Exposure and Transmission Applied to North East India
Source: PLoS One. 2012 Nov 27;7(11):e49713. doi: 10.1371/journal.pone.0049713 (PMC3507888; doi:10.1371/journal.pone.0049713)
Supplement: Table S1 — Coefficients of transmission and exposure for the 12 months. (DOC) [file pone.0049713.s007.doc]

| **Tirap** | | | | | | | | | | | | |
| --- | --- | --- | --- | --- | --- | --- | --- | --- | --- | --- | --- | --- |
| **Month** | **2006** | | **2007** | | **2008** | | **2009** | | **2010** | | **Average** | |
|  | **α_T_** | **α_E_** | **α_T_** | **α_E_** | **α_T_** | **α_E_** | **α_T_** | **α_E_** | **α_T_** | **α_E_** | **α_T_** | **α_E_** |
| **Jan** | 0.05 | 0.001 | 0.07 | 0.001 | 0.06 | 0.001 | 0.05 | 0.001 | 0.06 | 0.001 | 0.06 | 0.001 |
| **Feb** | 0.05 | 0.001 | 0.07 | 0.001 | 0.07 | 0.001 | 0.05 | 0.001 | 0.07 | 0.001 | 0.06 | 0.001 |
| **Mar** | 0.06 | 0.001 | 0.07 | 0.001 | 0.07 | 0.001 | 0.06 | 0.001 | 0.07 | 0.001 | 0.07 | 0.001 |
| **Apr** | 0.09 | 0.001 | 0.11 | 0.002 | 0.07 | 0.001 | 0.09 | 0.001 | 0.07 | 0.001 | 0.09 | 0.001 |
| **May** | 0.12 | 0.001 | 0.11 | 0.002 | 0.11 | 0.002 | 0.12 | 0.001 | 0.11 | 0.002 | 0.11 | 0.002 |
| **Jun** | 0.06 | 0.005 | 0.10 | 0.004 | 0.09 | 0.003 | 0.06 | 0.005 | 0.09 | 0.003 | 0.08 | 0.004 |
| **Jul** | 0.10 | 0.003 | 0.10 | 0.005 | 0.10 | 0.003 | 0.10 | 0.003 | 0.10 | 0.003 | 0.10 | 0.003 |
| **Aug** | 0.14 | 0.003 | 0.13 | 0.005 | 0.08 | 0.002 | 0.14 | 0.003 | 0.08 | 0.002 | 0.11 | 0.003 |
| **Sep** | 0.12 | 0.003 | 0.11 | 0.004 | 0.07 | 0.002 | 0.12 | 0.003 | 0.07 | 0.002 | 0.10 | 0.002 |
| **Oct** | 0.10 | 0.002 | NA | NA | 0.06 | 0.001 | 0.10 | 0.002 | 0.06 | 0.001 | 0.08 | 0.002 |
| **Nov** | 0.12 | 0.002 | NA | NA | 0.07 | 0.001 | 0.12 | 0.002 | 0.07 | 0.001 | 0.10 | 0.002 |
| **Dec** | 0.10 | 0.001 | NA | NA | 0.05 | 0.001 | 0.10 | 0.001 | 0.05 | 0.001 | 0.07 | 0.001 |
| **Avg** | **0.09** | **0.002** | **0.10** | **0.003** | **0.08** | **0.001** | **0.09** | **0.002** | **0.08** | **0.001** | **0.09** | **0.002** |

**Table S1 (a): Tirap**

| **Changlang** | | | | | | | | | | | | |
| --- | --- | --- | --- | --- | --- | --- | --- | --- | --- | --- | --- | --- |
| **Month** | **2006** | | **2007** | | **2008** | | **2009** | | **2010** | | **Average** | |
|  | **α_T_** | **α_E_** | **α_T_** | **α_E_** | **α_T_** | **α_E_** | **α_T_** | **α_E_** | **α_T_** | **α_E_** | **α_T_** | **α_E_** |
| **Jan** | 0.08 | 0.001 | 0.11 | 0.002 | 0.10 | 0.001 | 0.08 | 0.001 | 0.10 | 0.001 | 0.09 | 0.001 |
| **Feb** | 0.10 | 0.001 | 0.09 | 0.001 | 0.05 | 0.001 | 0.10 | 0.001 | 0.05 | 0.001 | 0.08 | 0.001 |
| **Mar** | 0.12 | 0.002 | 0.08 | 0.002 | 0.04 | 0.001 | 0.12 | 0.002 | 0.04 | 0.001 | 0.08 | 0.002 |
| **Apr** | 0.09 | 0.002 | 0.11 | 0.002 | 0.04 | 0.001 | 0.09 | 0.002 | 0.04 | 0.001 | 0.07 | 0.001 |
| **May** | 0.10 | 0.003 | 0.06 | 0.002 | 0.05 | 0.002 | 0.10 | 0.003 | 0.05 | 0.002 | 0.07 | 0.002 |
| **Jun** | 0.10 | 0.012 | 0.06 | 0.003 | 0.06 | 0.002 | 0.10 | 0.012 | 0.06 | 0.002 | 0.07 | 0.006 |
| **Jul** | 0.08 | 0.009 | 0.07 | 0.008 | 0.08 | 0.004 | 0.08 | 0.009 | 0.08 | 0.004 | 0.08 | 0.007 |
| **Aug** | 0.12 | 0.009 | 0.05 | 0.001 | 0.06 | 0.003 | 0.12 | 0.009 | 0.06 | 0.003 | 0.08 | 0.005 |
| **Sep** | 0.16 | 0.010 | 0.08 | 0.001 | 0.07 | 0.004 | 0.16 | 0.010 | 0.07 | 0.004 | 0.11 | 0.006 |
| **Oct** | 0.18 | 0.012 | 0.08 | 0.002 | 0.09 | 0.005 | 0.18 | 0.012 | 0.09 | 0.005 | 0.12 | 0.007 |
| **Nov** | 0.18 | 0.013 | NA | NA | 0.11 | 0.004 | 0.18 | 0.013 | 0.11 | 0.004 | 0.15 | 0.009 |
| **Dec** | 0.16 | 0.007 | NA | NA | 0.09 | 0.002 | 0.16 | 0.007 | 0.09 | 0.002 | 0.13 | 0.005 |
| **Avg** | **0.12** | **0.007** | **0.08** | **0.002** | **0.07** | **0.002** | **0.12** | **0.007** | **0.07** | **0.002** | **0.09** | **0.004** |

**Table S1 (b): Changlang.**

| **Lohit** | | | | | | | | | | | | |
| --- | --- | --- | --- | --- | --- | --- | --- | --- | --- | --- | --- | --- |
| **Month** | **2006** | | **2007** | | **2008** | | **2009** | | **2010** | | **Average** | |
|  | **α_T_** | **α_E_** | **α_T_** | **α_E_** | **α_T_** | **α_E_** | **α_T_** | **α_E_** | **α_T_** | **α_E_** | **α_T_** | **α_E_** |
| **Jan** | 0.05 | 0.001 | 0.07 | 0.001 | 0.08 | 0.001 | 0.05 | 0.001 | 0.08 | 0.001 | 0.06 | 0.001 |
| **Feb** | 0.05 | 0.001 | 0.06 | 0.001 | 0.09 | 0.001 | 0.05 | 0.001 | 0.09 | 0.001 | 0.07 | 0.001 |
| **Mar** | 0.08 | 0.002 | 0.08 | 0.002 | 0.08 | 0.001 | 0.08 | 0.002 | 0.08 | 0.001 | 0.08 | 0.001 |
| **Apr** | 0.10 | 0.002 | 0.89 | 0.002 | 0.06 | 0.001 | 0.10 | 0.002 | 0.06 | 0.001 | 0.24 | 0.002 |
| **May** | 0.10 | 0.005 | 0.12 | 0.007 | 0.07 | 0.002 | 0.10 | 0.005 | 0.07 | 0.002 | 0.09 | 0.004 |
| **Jun** | 0.11 | 0.012 | 0.13 | 0.014 | 0.10 | 0.004 | 0.11 | 0.012 | 0.10 | 0.004 | 0.11 | 0.009 |
| **Jul** | 0.12 | 0.017 | 0.12 | 0.019 | 0.13 | 0.006 | 0.12 | 0.017 | 0.13 | 0.006 | 0.12 | 0.013 |
| **Aug** | 0.13 | 0.010 | 0.12 | 0.009 | 0.13 | 0.005 | 0.13 | 0.010 | 0.13 | 0.005 | 0.13 | 0.008 |
| **Sep** | 0.12 | 0.008 | 0.16 | 0.009 | 0.12 | 0.005 | 0.12 | 0.008 | 0.12 | 0.005 | 0.13 | 0.007 |
| **Oct** | 0.13 | 0.007 | NA | NA | 0.13 | 0.004 | 0.13 | 0.007 | 0.13 | 0.004 | 0.13 | 0.006 |
| **Nov** | 0.17 | 0.007 | NA | NA | 0.13 | 0.003 | 0.17 | 0.007 | 0.13 | 0.003 | 0.15 | 0.005 |
| **Dec** | 0.11 | 0.003 | NA | NA | 0.11 | 0.002 | 0.11 | 0.003 | 0.11 | 0.002 | 0.11 | 0.002 |
| **Avg** | **0.11** | **0.006** | **0.19** | **0.007** | **0.10** | **0.003** | **0.11** | **0.006** | **0.10** | **0.003** | **0.12** | **0.005** |

**Table S1 (c):** Lohith

| **L/Dibang Valley** | | | | | | | | | | | | |
| --- | --- | --- | --- | --- | --- | --- | --- | --- | --- | --- | --- | --- |
| **Month** | **2006** | | **2007** | | **2008** | | **2009** | | **2010** | | **Average** | |
|  | **α_T_** | **α_E_** | **α_T_** | **α_E_** | **α_T_** | **α_E_** | **α_T_** | **α_E_** | **α_T_** | **α_E_** | **α_T_** | **α_E_** |
| **Jan** | 0.07 | 0.001 | 0.14 | 0.003 | 0.10 | 0.002 | 0.07 | 0.001 | 0.10 | 0.002 | 0.10 | 0.002 |
| **Feb** | 0.09 | 0.001 | 0.11 | 0.003 | 0.11 | 0.002 | 0.09 | 0.001 | 0.11 | 0.002 | 0.10 | 0.002 |
| **Mar** | 0.09 | 0.002 | 0.13 | 0.004 | 0.09 | 0.002 | 0.09 | 0.002 | 0.09 | 0.002 | 0.10 | 0.002 |
| **Apr** | 0.08 | 0.001 | 0.16 | 0.006 | 0.06 | 0.001 | 0.08 | 0.001 | 0.06 | 0.001 | 0.09 | 0.002 |
| **May** | 0.14 | 0.004 | 0.15 | 0.009 | 0.11 | 0.003 | 0.14 | 0.004 | 0.11 | 0.003 | 0.13 | 0.005 |
| **Jun** | 0.12 | 0.017 | 0.13 | 0.023 | 0.10 | 0.010 | 0.12 | 0.017 | 0.10 | 0.010 | 0.11 | 0.015 |
| **Jul** | 0.12 | 0.021 | 0.18 | 0.019 | 0.16 | 0.007 | 0.12 | 0.021 | 0.16 | 0.007 | 0.15 | 0.015 |
| **Aug** | 0.01 | 0.001 | 0.15 | 0.014 | 0.19 | 0.006 | 0.01 | 0.001 | 0.19 | 0.006 | 0.11 | 0.006 |
| **Sep** | 0.12 | 0.008 | 0.12 | 0.010 | 0.14 | 0.004 | 0.12 | 0.008 | 0.14 | 0.004 | 0.13 | 0.007 |
| **Oct** | 0.13 | 0.005 | NA | NA | 0.14 | 0.007 | 0.13 | 0.005 | 0.14 | 0.007 | 0.13 | 0.006 |
| **Nov** | 0.13 | 0.005 | NA | NA | 0.12 | 0.003 | 0.13 | 0.005 | 0.12 | 0.003 | 0.13 | 0.004 |
| **Dec** | 0.15 | 0.005 | NA | NA | 0.15 | 0.002 | 0.15 | 0.005 | 0.15 | 0.002 | 0.15 | 0.003 |
| **Avg** | **0.11** | **0.006** | **0.14** | **0.010** | **0.12** | **0.004** | **0.11** | **0.006** | **0.12** | **0.004** | **0.12** | **0.006** |

**Table S1 (d)**: Lower Dibang Valley

| **East Siangrecipita** | | | | | | | | | | | | |
| --- | --- | --- | --- | --- | --- | --- | --- | --- | --- | --- | --- | --- |
| **Month** | **2006** | | **2007** | | **2008** | | **2009** | | **2010** | | **Average** | |
|  | **α_T_** | **α_E_** | **α_T_** | **α_E_** | **α_T_** | **α_E_** | **α_T_** | **α_E_** | **α_T_** | **α_E_** | **α_T_** | **α_E_** |
| **Jan** | 0.14 | 0.004 | 0.16 | 0.004 | 0.11 | 0.003 | 0.14 | 0.004 | 0.11 | 0.003 | 0.13 | 0.004 |
| **Feb** | 0.13 | 0.003 | 0.15 | 0.006 | 0.14 | 0.004 | 0.13 | 0.003 | 0.14 | 0.004 | 0.14 | 0.004 |
| **Mar** | 0.12 | 0.005 | 0.14 | 0.006 | 0.13 | 0.004 | 0.12 | 0.005 | 0.13 | 0.004 | 0.13 | 0.005 |
| **Apr** | 0.15 | 0.005 | 0.15 | 0.006 | 0.15 | 0.006 | 0.15 | 0.005 | 0.15 | 0.006 | 0.15 | 0.006 |
| **May** | 0.13 | 0.009 | 0.16 | 0.011 | 0.15 | 0.008 | 0.13 | 0.009 | 0.15 | 0.008 | 0.14 | 0.009 |
| **Jun** | 0.18 | 0.014 | 0.17 | 0.015 | 0.06 | 0.003 | 0.18 | 0.014 | 0.06 | 0.003 | 0.13 | 0.010 |
| **Jul** | 0.19 | 0.025 | 0.15 | 0.020 | 0.15 | 0.024 | 0.19 | 0.025 | 0.15 | 0.024 | 0.17 | 0.023 |
| **Aug** | 0.17 | 0.019 | 0.16 | 0.017 | 0.19 | 0.021 | 0.17 | 0.019 | 0.19 | 0.021 | 0.18 | 0.019 |
| **Sep** | 0.18 | 0.009 | 0.14 | 0.009 | 0.16 | 0.016 | 0.18 | 0.009 | 0.16 | 0.016 | 0.16 | 0.012 |
| **Oct** | 0.18 | 0.008 | NA | NA | 0.15 | 0.012 | 0.18 | 0.008 | 0.15 | 0.012 | 0.17 | 0.010 |
| **Nov** | 0.18 | 0.007 | NA | NA | 0.16 | 0.009 | 0.18 | 0.007 | 0.16 | 0.009 | 0.17 | 0.008 |
| **Dec** | 0.19 | 0.004 | NA | NA | 0.10 | 0.004 | 0.19 | 0.004 | 0.10 | 0.004 | 0.15 | 0.004 |
| **Avg** | **0.16** | **0.009** | **0.15** | **0.010** | **0.14** | **0.010** | **0.16** | **0.009** | **0.14** | **0.010** | **0.15** | **0.010** |

**Table S1 (e):** East Siang

| **West Siang** | | | | | | | | | | | | |
| --- | --- | --- | --- | --- | --- | --- | --- | --- | --- | --- | --- | --- |
| **Month** | **2006** | | **2007** | | **2008** | | **2009** | | **2010** | | **Average** | |
|  | **α_T_** | **α_E_** | **α_T_** | **α_E_** | **α_T_** | **α_E_** | **α_T_** | **α_E_** | **α_T_** | **α_E_** | **α_T_** | **α_E_** |
| **Jan** | 0.12 | 0.001 | 0.08 | 0.001 | 0.16 | 0.001 | 0.12 | 0.001 | 0.16 | 0.001 | 0.13 | 0.001 |
| **Feb** | 0.16 | 0.001 | 0.12 | 0.001 | 0.16 | 0.001 | 0.16 | 0.001 | 0.16 | 0.001 | 0.15 | 0.001 |
| **Mar** | 0.18 | 0.002 | 0.21 | 0.001 | 0.19 | 0.002 | 0.18 | 0.002 | 0.19 | 0.002 | 0.19 | 0.002 |
| **Apr** | 0.18 | 0.002 | 0.21 | 0.002 | 0.20 | 0.002 | 0.18 | 0.002 | 0.20 | 0.002 | 0.19 | 0.002 |
| **May** | 0.21 | 0.004 | 0.36 | 0.004 | 0.23 | 0.004 | 0.21 | 0.004 | 0.23 | 0.004 | 0.25 | 0.004 |
| **Jun** | 0.20 | 0.007 | 0.42 | 0.004 | 0.19 | 0.006 | 0.20 | 0.007 | 0.19 | 0.006 | 0.24 | 0.006 |
| **Jul** | 0.19 | 0.011 | 0.20 | 0.011 | 0.15 | 0.002 | 0.19 | 0.011 | 0.15 | 0.002 | 0.18 | 0.007 |
| **Aug** | 0.19 | 0.004 | 0.27 | 0.007 | 0.25 | 0.006 | 0.19 | 0.004 | 0.25 | 0.006 | 0.23 | 0.005 |
| **Sep** | 0.10 | 0.002 | 0.21 | 0.005 | 0.32 | 0.007 | 0.10 | 0.002 | 0.32 | 0.007 | 0.21 | 0.005 |
| **Oct** | 0.10 | 0.001 | NA | NA | 0.22 | 0.004 | 0.10 | 0.001 | 0.22 | 0.004 | 0.16 | 0.002 |
| **Nov** | 0.09 | 0.001 | NA | NA | 0.21 | 0.003 | 0.09 | 0.001 | 0.21 | 0.003 | 0.15 | 0.002 |
| **Dec** | 0.11 | 0.001 | NA | NA | 0.13 | 0.002 | 0.11 | 0.001 | 0.13 | 0.002 | 0.12 | 0.001 |
| **Avg** | **0.15** | **0.003** | **0.23** | **0.004** | **0.20** | **0.003** | **0.15** | **0.003** | **0.20** | **0.003** | **0.19** | **0.003** |

**Table S1 (f):** West Siang

| **Upper Siang** | | | | | | | | | | | | |
| --- | --- | --- | --- | --- | --- | --- | --- | --- | --- | --- | --- | --- |
| **Month** | **2006** | | **2007** | | **2008** | | **2009** | | **2010** | | **Average** | |
|  | **α_T_** | **α_E_** | **α_T_** | **α_E_** | **α_T_** | **α_E_** | **α_T_** | **α_E_** | **α_T_** | **α_E_** | **α_T_** | **α_E_** |
| **Jan** | 0.05 | 0.001 | 0.03 | 0.001 | 0.10 | 0.001 | 0.05 | 0.001 | 0.10 | 0.001 | 0.07 | 0.001 |
| **Feb** | 0.05 | 0.001 | 0.04 | 0.001 | 0.09 | 0.001 | 0.05 | 0.001 | 0.09 | 0.001 | 0.06 | 0.001 |
| **Mar** | 0.08 | 0.001 | 0.11 | 0.001 | 0.08 | 0.001 | 0.08 | 0.001 | 0.08 | 0.001 | 0.09 | 0.001 |
| **Apr** | 0.07 | 0.001 | 0.09 | 0.001 | 0.05 | 0.002 | 0.07 | 0.001 | 0.05 | 0.002 | 0.07 | 0.001 |
| **May** | 0.12 | 0.001 | 0.14 | 0.001 | 0.10 | 0.001 | 0.12 | 0.001 | 0.10 | 0.001 | 0.11 | 0.001 |
| **Jun** | 0.10 | 0.002 | 0.14 | 0.002 | 0.10 | 0.002 | 0.10 | 0.002 | 0.10 | 0.002 | 0.11 | 0.002 |
| **Jul** | NA | NA | 0.12 | 0.002 | 0.16 | 0.003 | 0.09 | 0.001 | 0.16 | 0.003 | 0.13 | 0.002 |
| **Aug** | NA | NA | 0.08 | 0.001 | 0.08 | 0.003 | 0.11 | 0.001 | 0.08 | 0.003 | 0.09 | 0.002 |
| **Sep** | NA | NA | NA | NA | 0.07 | 0.002 | 0.08 | 0.001 | 0.07 | 0.002 | 0.07 | 0.002 |
| **Oct** | NA | NA | NA | NA | 0.09 | 0.002 | 0.07 | 0.001 | 0.09 | 0.002 | 0.08 | 0.001 |
| **Nov** | NA | NA | NA | NA | 0.06 | 0.001 | 0.12 | 0.001 | 0.06 | 0.001 | 0.08 | 0.001 |
| **Dec** | NA | NA | NA | NA | 0.12 | 0.001 | 0.11 | 0.002 | 0.12 | 0.001 | 0.12 | 0.001 |
| **Avg** | **0.08** | **0.001** | **0.09** | **0.001** | **0.09** | **0.002** | **0.09** | **0.001** | **0.09** | **0.002** | **0.09** | **0.001** |

**Table S1 (g):** Upper Siang

| **Upper Subansiri** | | | | | | | | | | | | |
| --- | --- | --- | --- | --- | --- | --- | --- | --- | --- | --- | --- | --- |
| **Month** | **2006** | | **2007** | | **2008** | | **2009** | | **2010** | | **Average** | |
|  | **α_T_** | **α_E_** | **α_T_** | **α_E_** | **α_T_** | **α_E_** | **α_T_** | **α_E_** | **α_T_** | **α_E_** | **α_T_** | **α_E_** |
| **Jan** | 0.24 | 0.001 | 0.25 | 0.001 | 0.24 | 0.001 | 0.24 | 0.001 | 0.24 | 0.001 | 0.24 | 0.001 |
| **Feb** | 0.32 | 0.004 | 0.20 | 0.001 | 0.27 | 0.001 | 0.32 | 0.004 | 0.27 | 0.001 | 0.28 | 0.002 |
| **Mar** | 0.13 | 0.001 | 0.21 | 0.001 | 0.24 | 0.001 | 0.13 | 0.001 | 0.24 | 0.001 | 0.19 | 0.001 |
| **Apr** | 0.18 | 0.002 | 0.16 | 0.001 | 0.27 | 0.001 | 0.18 | 0.002 | 0.27 | 0.001 | 0.21 | 0.001 |
| **May** | 0.11 | 0.002 | 0.17 | 0.001 | 0.26 | 0.001 | 0.11 | 0.002 | 0.26 | 0.001 | 0.18 | 0.001 |
| **Jun** | 0.11 | 0.004 | 0.17 | 0.001 | 0.30 | 0.002 | 0.11 | 0.004 | 0.30 | 0.002 | 0.20 | 0.003 |
| **Jul** | 0.12 | 0.008 | 0.26 | 0.005 | 0.26 | 0.002 | 0.12 | 0.008 | 0.26 | 0.002 | 0.21 | 0.005 |
| **Aug** | 0.12 | 0.003 | 0.16 | 0.002 | 0.23 | 0.003 | 0.12 | 0.003 | 0.23 | 0.003 | 0.17 | 0.003 |
| **Sep** | 0.19 | 0.004 | 0.18 | 0.001 | 0.27 | 0.004 | 0.19 | 0.004 | 0.27 | 0.004 | 0.22 | 0.003 |
| **Oct** | 0.21 | 0.005 | 0.21 | 0.002 | 0.19 | 0.001 | 0.21 | 0.005 | 0.19 | 0.001 | 0.20 | 0.003 |
| **Nov** | 0.16 | 0.002 | NA | NA | 0.25 | 0.001 | 0.16 | 0.002 | 0.25 | 0.001 | 0.20 | 0.002 |
| **Dec** | 0.18 | 0.002 | NA | NA | 0.18 | 0.001 | 0.18 | 0.002 | 0.18 | 0.001 | 0.18 | 0.001 |
| **Avg** | **0.17** | **0.003** | **0.20** | **0.002** | **0.25** | **0.002** | **0.17** | **0.003** | **0.25** | **0.002** | **0.21** | **0.002** |

**Table S1 (h):** Upper Subansiri

| **Papum Pare** | | | | | | | | | | | | |
| --- | --- | --- | --- | --- | --- | --- | --- | --- | --- | --- | --- | --- |
| **Month** | **2006** | | **2007** | | **2008** | | **2009** | | **2010** | | **Average** | |
|  | **α_T_** | **α_E_** | **α_T_** | **α_E_** | **α_T_** | **α_E_** | **α_T_** | **α_E_** | **α_T_** | **α_E_** | **α_T_** | **α_E_** |
| **Jan** | 0.19 | 0.004 | 0.08 | 0.002 | 0.05 | 0.001 | 0.19 | 0.004 | 0.05 | 0.001 | 0.11 | 0.003 |
| **Feb** | 0.18 | 0.003 | 0.07 | 0.002 | 0.06 | 0.001 | 0.18 | 0.003 | 0.06 | 0.001 | 0.11 | 0.002 |
| **Mar** | 0.14 | 0.005 | 0.04 | 0.001 | 0.08 | 0.001 | 0.14 | 0.005 | 0.08 | 0.001 | 0.10 | 0.003 |
| **Apr** | 0.16 | 0.010 | 0.10 | 0.003 | 0.07 | 0.002 | 0.16 | 0.010 | 0.07 | 0.002 | 0.11 | 0.005 |
| **May** | 0.17 | 0.010 | 0.14 | 0.005 | 0.08 | 0.003 | 0.17 | 0.010 | 0.08 | 0.003 | 0.13 | 0.006 |
| **Jun** | 0.15 | 0.010 | 0.12 | 0.008 | 0.10 | 0.004 | 0.15 | 0.010 | 0.10 | 0.004 | 0.12 | 0.007 |
| **Jul** | 0.14 | 0.012 | 0.14 | 0.013 | 0.09 | 0.004 | 0.14 | 0.012 | 0.09 | 0.004 | 0.12 | 0.009 |
| **Aug** | 0.13 | 0.007 | 0.11 | 0.008 | 0.09 | 0.004 | 0.13 | 0.007 | 0.09 | 0.004 | 0.11 | 0.006 |
| **Sep** | 0.13 | 0.006 | 0.11 | 0.006 | 0.09 | 0.003 | 0.13 | 0.006 | 0.09 | 0.003 | 0.11 | 0.005 |
| **Oct** | 0.18 | 0.010 | 0.07 | 0.004 | 0.09 | 0.003 | 0.18 | 0.010 | 0.09 | 0.003 | 0.12 | 0.006 |
| **Nov** | 0.16 | 0.006 | NA | NA | 0.10 | 0.002 | 0.16 | 0.006 | 0.10 | 0.002 | 0.13 | 0.004 |
| **Dec** | 0.13 | 0.003 | NA | NA | 0.06 | 0.001 | 0.13 | 0.003 | 0.06 | 0.001 | 0.09 | 0.002 |
| **Avg** | **0.15** | **0.007** | **0.10** | **0.005** | **0.08** | **0.002** | **0.15** | **0.007** | **0.08** | **0.002** | **0.11** | **0.005** |

**Table S1 (i):** Papum Pare

| **K/Kumey** | | | | | | | | | | | | |
| --- | --- | --- | --- | --- | --- | --- | --- | --- | --- | --- | --- | --- |
| **Month** | **2006** | | **2007** | | **2008** | | **2009** | | **2010** | | **Average** | |
|  | **α_T_** | **α_E_** | **α_T_** | **α_E_** | **α_T_** | **α_E_** | **α_T_** | **α_E_** | **α_T_** | **α_E_** | **α_T_** | **α_E_** |
| **Jan** | 0.37 | 0.001 | 1.00 | 0.001 | 0.81 | 0.001 | 0.37 | 0.001 | 0.81 | 0.001 | 0.67 | 0.001 |
| **Feb** | 0.31 | 0.001 | 0.98 | 0.000 | 0.76 | 0.001 | 0.31 | 0.001 | 0.76 | 0.001 | 0.62 | 0.001 |
| **Mar** | 0.14 | 0.001 | 1.00 | 0.001 | 0.72 | 0.001 | 0.14 | 0.001 | 0.72 | 0.001 | 0.55 | 0.001 |
| **Apr** | 0.16 | 0.001 | 0.99 | 0.001 | 0.93 | 0.001 | 0.16 | 0.001 | 0.93 | 0.001 | 0.64 | 0.001 |
| **May** | 0.10 | 0.001 | 1.00 | 0.001 | 0.97 | 0.002 | 0.10 | 0.001 | 0.97 | 0.002 | 0.63 | 0.001 |
| **Jun** | 0.10 | 0.001 | 0.98 | 0.001 | 0.97 | 0.002 | 0.10 | 0.001 | 0.97 | 0.002 | 0.62 | 0.001 |
| **Jul** | 0.10 | 0.001 | 1.00 | 0.001 | 1.02 | 0.002 | 0.10 | 0.001 | 1.02 | 0.002 | 0.65 | 0.002 |
| **Aug** | 0.10 | 0.001 | 0.98 | 0.001 | 0.80 | 0.002 | 0.10 | 0.001 | 0.80 | 0.002 | 0.56 | 0.002 |
| **Sep** | 0.10 | 0.001 | 0.96 | 0.001 | 0.81 | 0.003 | 0.10 | 0.001 | 0.81 | 0.003 | 0.56 | 0.002 |
| **Oct** | 0.16 | 0.002 | 0.99 | 0.001 | 0.86 | 0.003 | 0.16 | 0.002 | 0.86 | 0.003 | 0.60 | 0.002 |
| **Nov** | 0.36 | 0.001 | NA | NA | 0.95 | 0.001 | 0.36 | 0.001 | 0.95 | 0.001 | 0.66 | 0.001 |
| **Dec** | 0.19 | 0.001 | NA | NA | 0.81 | 0.001 | 0.19 | 0.001 | 0.81 | 0.001 | 0.50 | 0.001 |
| **Avg** | **0.18** | **0.001** | **0.99** | **0.001** | **0.87** | **0.002** | **0.18** | **0.001** | **0.87** | **0.002** | **0.62** | **0.001** |

**Table S1 (j):** Kurung Kume

| **East Kameng** | | | | | | | | | | | | |
| --- | --- | --- | --- | --- | --- | --- | --- | --- | --- | --- | --- | --- |
| **Month** | **2006** | | **2007** | | **2008** | | **2009** | | **2010** | | **Average** | |
|  | **α_T_** | **α_E_** | **α_T_** | **α_E_** | **α_T_** | **α_E_** | **α_T_** | **α_E_** | **α_T_** | **α_E_** | **α_T_** | **α_E_** |
| **Jan** | 0.21 | 0.001 | 0.24 | 0.004 | 0.10 | 0.001 | 0.21 | 0.001 | 0.10 | 0.001 | 0.17 | 0.002 |
| **Feb** | 0.14 | 0.001 | 0.25 | 0.003 | 0.21 | 0.001 | 0.14 | 0.001 | 0.21 | 0.001 | 0.19 | 0.002 |
| **Mar** | 0.20 | 0.002 | 0.27 | 0.004 | 0.27 | 0.002 | 0.20 | 0.002 | 0.27 | 0.002 | 0.24 | 0.002 |
| **Apr** | 0.21 | 0.003 | 0.29 | 0.010 | 0.19 | 0.004 | 0.21 | 0.003 | 0.19 | 0.004 | 0.22 | 0.005 |
| **May** | 0.29 | 0.005 | 0.34 | 0.022 | 0.35 | 0.005 | 0.29 | 0.005 | 0.35 | 0.005 | 0.32 | 0.009 |
| **Jun** | 0.23 | 0.009 | 0.41 | 0.018 | 0.30 | 0.004 | 0.23 | 0.009 | 0.30 | 0.004 | 0.29 | 0.009 |
| **Jul** | 0.23 | 0.005 | 0.23 | 0.006 | 0.32 | 0.017 | 0.23 | 0.005 | 0.32 | 0.017 | 0.26 | 0.010 |
| **Aug** | 0.19 | 0.003 | 0.21 | 0.006 | 0.26 | 0.047 | 0.19 | 0.003 | 0.26 | 0.047 | 0.22 | 0.021 |
| **Sep** | 0.16 | 0.001 | 0.20 | 0.002 | 0.39 | 0.046 | 0.16 | 0.001 | 0.39 | 0.046 | 0.26 | 0.019 |
| **Oct** | 0.69 | 0.014 | 0.22 | 0.003 | 0.09 | 0.001 | 0.69 | 0.014 | 0.09 | 0.001 | 0.36 | 0.007 |
| **Nov** | 0.53 | 0.024 | NA | NA | 0.25 | 0.004 | 0.53 | 0.024 | 0.25 | 0.004 | 0.39 | 0.014 |
| **Dec** | 0.82 | 0.004 | NA | NA | 0.21 | 0.001 | 0.82 | 0.004 | 0.21 | 0.001 | 0.51 | 0.003 |
| **Avg** | **0.33** | **0.006** | **0.27** | **0.008** | **0.24** | **0.011** | **0.33** | **0.006** | **0.24** | **0.011** | **0.28** | **0.008** |

**Table S1 (k):** East Kameng

| **West Kameng** | | | | | | | | | | | | |
| --- | --- | --- | --- | --- | --- | --- | --- | --- | --- | --- | --- | --- |
| **Month** | **2006** | | **2007** | | **2008** | | **2009** | | **2010** | | **Average** | |
|  | **α_T_** | **α_E_** | **α_T_** | **α_E_** | **α_T_** | **α_E_** | **α_T_** | **α_E_** | **α_T_** | **α_E_** | **α_T_** | **α_E_** |
| **Jan** | 0.04 | 0.001 | 0.02 | 0.001 | 0.03 | 0.001 | 0.04 | 0.001 | 0.03 | 0.001 | 0.03 | 0.001 |
| **Feb** | 0.01 | 0.001 | 0.02 | 0.001 | 0.04 | 0.001 | 0.01 | 0.001 | 0.04 | 0.001 | 0.03 | 0.001 |
| **Mar** | 0.10 | 0.001 | 0.02 | 0.001 | 0.03 | 0.001 | 0.10 | 0.001 | 0.03 | 0.001 | 0.06 | 0.001 |
| **Apr** | 0.10 | 0.001 | 0.12 | 0.001 | 0.03 | 0.001 | 0.10 | 0.001 | 0.03 | 0.001 | 0.08 | 0.001 |
| **May** | 0.08 | 0.001 | 0.06 | 0.001 | 0.04 | 0.001 | 0.08 | 0.001 | 0.04 | 0.001 | 0.06 | 0.001 |
| **Jun** | 0.10 | 0.002 | 0.07 | 0.001 | 0.06 | 0.001 | 0.10 | 0.002 | 0.06 | 0.001 | 0.08 | 0.001 |
| **Jul** | 0.07 | 0.002 | 0.06 | 0.001 | 0.02 | 0.001 | 0.07 | 0.002 | 0.02 | 0.001 | 0.05 | 0.001 |
| **Aug** | 0.14 | 0.002 | 0.03 | 0.001 | 0.01 | 0.001 | 0.14 | 0.002 | 0.01 | 0.001 | 0.07 | 0.002 |
| **Sep** | 0.08 | 0.001 | NA | NA | 0.05 | 0.001 | 0.08 | 0.001 | 0.05 | 0.001 | 0.07 | 0.001 |
| **Oct** | 0.06 | 0.001 | NA | NA | 0.04 | 0.001 | 0.06 | 0.001 | 0.04 | 0.001 | 0.05 | 0.001 |
| **Nov** | 0.10 | 0.001 | NA | NA | 0.07 | 0.001 | 0.10 | 0.001 | 0.07 | 0.001 | 0.08 | 0.001 |
| **Dec** | 0.09 | 0.001 | NA | NA | 0.03 | 0.001 | 0.09 | 0.001 | 0.03 | 0.001 | 0.06 | 0.001 |
| **Avg** | **0.08** | **0.001** | **0.05** | **0.001** | **0.04** | **0.001** | **0.08** | **0.001** | **0.04** | **0.001** | **0.06** | **0.001** |

**Table S1 (l):** West Kameng
